# Epitope-specific antibodies can distinguish between soluble huntingtin exon-1 and its diverse cellular aggregates

**DOI:** 10.1016/j.jbc.2025.111048

**Published:** 2025-12-12

**Authors:** Joshua Lugo, Hui Xu, Jeannie Chen, Ali Khoshnan, Ralf Langen

**Affiliations:** Department of Physiology and Neuroscience and Zilkha Neurogenetic Institute, Keck School of Medicine, University of Southern California, Los Angeles, California, USA

**Keywords:** Huntington's disease, protein aggregation, biomarker, electron paramagnetic resonance, polyglutamine, antibody

## Abstract

Misfolding and aggregation of huntingtin exon-1 (Httex1) with an expanded polyglutamine region is a key pathological hallmark of Huntington’s disease, making conformationally specific Httex1 binders potentially valuable diagnostic or therapeutic tools. To define epitopes, which might confer conformationally specific Httex1 binding, we characterized five newly developed huntingtin antibodies (PHP5–PHP9). Binding to recombinant proteins as well as staining of human embryonic kidney 293 cells and R6/1 mice shows that PHP5 and PHP6 preferentially bind monomers over fibrils. Using electron paramagnetic resonance, peptide arrays, and deletion mutants, we mapped binding of PHP5 and PHP6 to the hydrophobic surface of an N-terminal α-helix spanning residues 4 to 18 of Httex1. In contrast, PHP7, PHP8, and PHP9, raised against protofibrils, recognize proline repeats within the C-terminal proline-rich domain (PRD). These antibodies showed a preference for aggregates in cells, but neither the N-terminal N17 region nor the polyglutamine fibril–forming core region was required. Similar fibril binding was also observed with an α-synuclein–PRD chimera, where the PRD was fused to the fibril-forming core of α-synuclein. Thus, a high density of PRD regions, rather than fibril core features, is needed for fibril binding. Interestingly, all PRD-binding antibodies, including PHP1 and P90, preferentially bound aggregates, but recognition of different cellular aggregates varied, revealing heterogeneity both within aggregates (rim *versus* interior) and between aggregates. Together, the binding principles uncovered here could serve as a basis for the design and optimization of binders with potential diagnostic or therapeutic relevance.

Huntington's disease (HD) is a progressive neurodegenerative disorder that affects movement control, cognition, and behavior (1). HD is caused by an expanded CAG trinucleotide repeat in the HTT gene, leading to the formation of mutant huntingtin protein with an abnormally long polyglutamine (polyQ) region (2). These polyQ expansions make N-terminal fragments of mutant huntingtin more prone to aggregation, resulting in the formation of fibrils and aggregates in the brain (3, 4). N-terminal fragments of huntingtin can be generated by proteolysis or aberrant splicing (5, 6). The role of these fragments in disease is supported by findings that expression of the first exon 1 of mutant huntingtin (Httex1) leads to aggregate formation and disease phenotypes in cell and animal models of HD (4).

Httex1 has three main domains (Fig. 1*A*); an N-terminal 17-amino acid region (N17), a Gln repeat region (polyQ) of variable length, and a proline-rich C-terminal domain (PRD). The latter can be broken down into two perfect proline repeat regions (P1 and P2) and two additional proline-containing regions (L17 and C-ter, Fig. 1*A*). When first generated, Httex1 is predominantly monomeric with a highly dynamic structure, where the N17 region and the N-terminal portion of the polyQ are partially α-helical, whereas the PRD is highly extended because of significant polyproline-II helical structure (7, 8, 9). The N17 and polyQ regions both undergo conformational changes upon oligomerization and aggregation. The N17 region is thought to play an early role in Httex1 aggregation by promoting the formation of dimers, tetramers, and larger oligomeric species (10, 11, 12, 13). These oligomers bring multiple polyQ regions together, which, in turn, promotes a structural transition of the polyQ region into the β-sheet containing core of Httex1 fibrils (14, 15, 16). Unlike the N17 and polyQ regions, the PRD does not undergo significant structural changes upon aggregation (17, 18). According to the bottle brush model, the PRD radiates outward from the fibril core by mainly forming polyproline 2 helical structure, and this region is generally thought to slow down aggregation (18, 19, 20).

**Figure 1:**
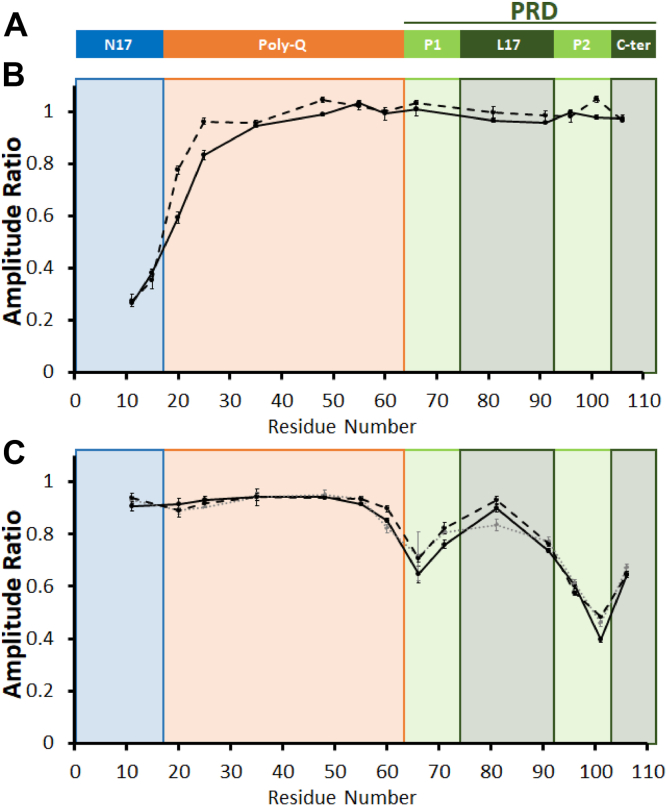
**Domain organization of Httex1 and involvement of individual domains in PHP5–PHP9 binding**. *A*, schematic illustration of the Httex1 domain organization. *B* and *C*, illustrate the involvement of different domains in the binding of PHP5–PHP6 and PHP7–PHP9, respectively. Local binding interactions were quantified from the ratio of the EPR intensities of singly spin-labeled Trx-Httex1(Q46) derivatives (10 μM) in the presence or the absence of the different antibodies (5 μM) for each labeling position (indicated on the *x*-axis). The EPR spectra were obtained in triplicate, and representative spectra are shown in Figure S1. PHP5 and PHP6 in *B* are represented by *dashed* and *solid lines*, respectively, whereas PHP7, PHP8, and PHP9 in *C* are represented by *solid black*, *dotted gray*, and *dashed black lines*, respectively. Points and bars indicate the mean ± standard deviation of triplicate measurements. EPR, electron paramagnetic resonance; Httex1, huntingtin exon-1.

Considering the importance of protein aggregation in HD and other neurodegenerative diseases, numerous strategies, including immunotherapies, have been explored for preventing aggregation or for clearing existing aggregates. Importantly, in the case of Alzheimer’s disease, such antibodies have been found to be beneficial in mouse models, and some have already been approved for the treatment of Alzheimer’s disease in humans, where they help to clear Aβ aggregates (21). In addition, antibodies have long been used for detecting specific proteins. Thus, antibodies and other binders can be of therapeutic as well as diagnostic use. Conformational specificity can be important in both contexts. In addition to merely detecting the presence of conformers, conformationally specific binders could be used for inhibiting aggregation, promoting clearance, or facilitating degradation. We have recently developed a series of antibodies raised against protofibrillar Httex1 (PHP7, PHP8, and PHP9) or short peptides containing the N17 region and the adjacent seven Gln residues (PHP5 and PHP6) (22). However, the precise epitopes, the modes of binding, and the potential conformational specificity of these antibodies remain unknown. Filling these knowledge gaps provides a first step for evaluating the diagnostic or therapeutic value of these antibodies.

Here, we used a combination of biophysical and biochemical methods to investigate the molecular mechanism by which PHP5–PHP9 bind to different Httex1 conformers. Our results indicate that PHP5 and PHP6 bind to the hydrophobic surface of an α-helix formed by the N17 region and the first Gln residue, roughly encompassing residues 4 to 18. These antibodies readily bind to monomeric Httex1, whereas their reactivity to fibrillar and aggregated protein is reduced in dot blots and cells. PHP7–PHP9 recognize the PRD, but, unlike other fibril binding antibodies that also bind to the PRD (PHP1, PHP2, MW8, and P90 (23, 24, 25)), they mainly recognize the perfect Pro repeat regions. PHP7–PHP9 bind to monomers, but their most pronounced interaction is with fibrils. PHP7–PHP9, like all other PRD binding antibodies, recognize a region of the protein that does not undergo major conformational changes during fibrilization. Binding to fibrils could be observed even when the N17 and polyQ regions of Httex1 were replaced with the fibril-forming core region of α-synuclein. Thus, PRD binding antibodies are not sensors of fibril structure *per se*; rather, these antibodies largely sense the enhanced bristle densities in aggregates. Intriguingly, the set of PRD antibodies can be used to differentiate between different types of aggregates in cells, where they reveal heterogeneity within and between aggregates.

## Results

Antibody binding to monomeric Httex1 can readily be detected by electron paramagnetic resonance (EPR), as the Httex1 monomer is highly dynamic, giving rise to sharp and narrowly spaced EPR lines of high amplitude (7, 26). When a spin-labeled residue is at or near a binding site, its mobility becomes reduced, which in turn leads to a broadening of the EPR lines and a reduction in amplitude. We have recently used this approach to determine the binding regions in Httex1 for MW1 (26) and it has also been applied to the study of rhodopsin antibody interactions (27). As in our prior study (26), we used the less aggregation-prone thioredoxin fusion protein of Httex1(Q46), Trx-Httex1(Q46), which increases the stability of the monomeric protein. Several singly spin-labeled derivatives were generated with spin labels at selected sites in the N17, polyQ, and PRD regions. In the case of PHP5 and PHP6, noticeable spectral changes were observed for sites in the N17 region (11R1 and 15R1; Fig. S1*A*), whereas sites in the polyQ and PRD were not strongly impacted. As a control, we performed dot blot experiments and found that the introduction of spin labels did not interfere with binding (Fig. S2). PHP7, PHP8, and PHP9 caused the strongest spectral changes at sites in the PRD (positions 66, 91, 96, and 101, Fig. S1*B*). Again, binding at sites outside this region was verified by dot blots (Fig. S2).

To quantify the effects of antibody binding, we plotted the relative amplitudes, which represent the ratio of the EPR intensities obtained in the presence or absence of antibody (26). As shown in Figure 1*B*, the strongest amplitude reductions for PHP5 and PHP6 binding are localized to residues in the N17 region. In contrast, PHP7, PHP8, and PHP9 exhibited the largest spectral changes in the two perfect Pro repeats (P1 and P2) of the PRD (Fig. 1, *A* and *C*). We also note apparent mobility gradients for all antibody binding interactions. For example, in the case of PHP5 and PHP6, there is some immobilization that reaches into the polyQ region (Fig. 1*B*). Such mobility gradients are commonly observed in highly dynamic proteins, where structural ordering caused by binding can affect the mobility of residues that are up to 10 amino acids away in sequence (27, 28). Similar considerations also apply to the L17 region, where the reduction of mobility was likely caused indirectly by the proximity to P1 and P2 (Fig. 1*C*, also see below).

**Figure 2:**
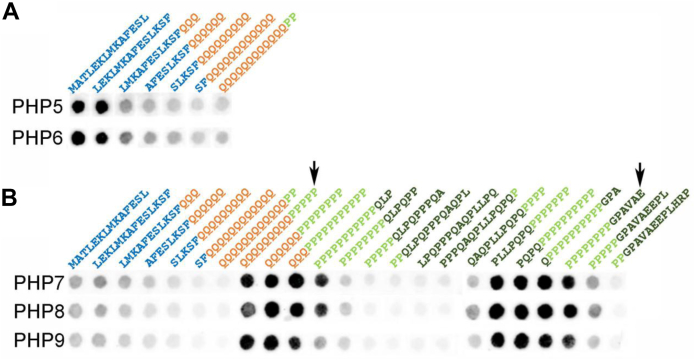
**Dot blot of PHP5–PHP9 binding to 14-residue peptide fragments of Httex1**. The binding of PHP5, PHP6, and PHP7–PHP9 to peptides containing overlapping 14 residues of Httex1 is shown in *A* and *B*, respectively. The color coding of the sequences is as in Figure 1 and indicates the regions to which the peptide sequences correspond. For PHP5 and PHP6, only binding to N17 and polyQ sequences was tried, as these antibodies were raised against an N17Q7 peptide. The *arrows* point to peptides that contain consecutive proline residues but which have flanking regions without any sequence or amino acid similarities, indicating that consecutive proline residues are the primary driver of binding. Httex1, huntingtin exon-1.

To further test the conclusions from our EPR experiments, we examined the binding of PHP5–PHP9 to peptide strips and deletion mutants. The peptide strips contained 14-amino acid long peptides that correspond to different regions of the Httex1 sequence (Fig. 2). PHP5 and PHP6 exhibited the strongest binding to peptides derived from the N17 sequences, such as residues 1 to 14 or residues 4 to 17, whereas binding was reduced in subsequent peptides where the N17 residues were successively replaced with glutamines (Fig. 2*A*). Moreover, a Trx-Httex1(Q46) truncation mutant lacking the N17 region (ΔN17), but not a mutant lacking the PRD (ΔPRD), resulted in a loss of binding in dot blots (Fig. S3). Together, these data further support the notion that PHP5 and PHP6 target the N17 region.

**Figure 3:**
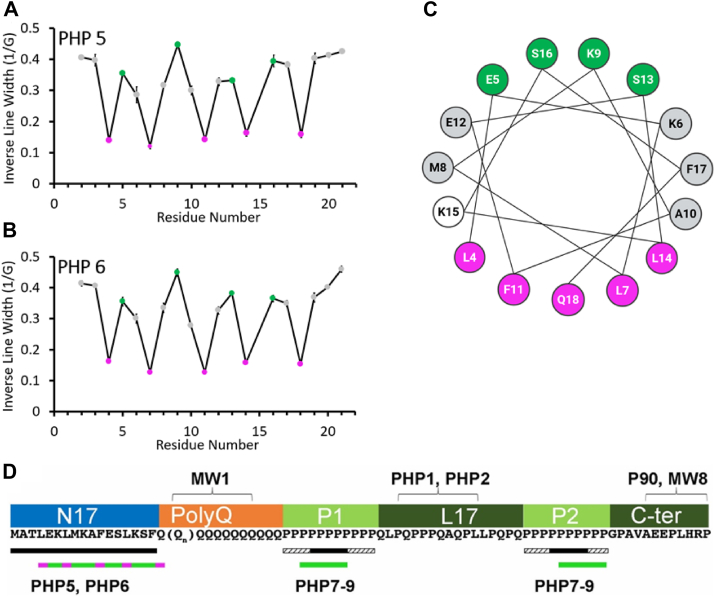
**PHP5 and PHP6 bind to the hydrophobic surface of an amphipathic helix formed by N17 residues and Gln18**. Nitroxide spin labeling was used to map binding interactions of PHP5 and PHP6 with untagged Httex1(Q25) monomers. EPR spectra of spin-labeled derivatives in the antibody-bound state (Fig. S4) were analyzed to determine site-specific immobilization. The inverse of the central EPR line width, a well-established mobility parameter, was plotted as a function of labeling position, revealing periodic oscillations for PHP5 (*A*) and PHP6 (*B*). These oscillations are consistent with an α-helical structure, where the maxima (highest mobility, *green*) and minima (lowest mobility, *magenta*) fall onto opposing surfaces of a helical wheel (*C*). The remaining residues are shaded *gray*, except for K15, which is shown in *white* since it was not tested. These data indicate that PHP5 and PHP6 interact with the hydrophobic surface of an amphipathic helix formed by the N17 region as well as Gln18. Data were obtained in triplicate. A summary of the EPR and peptide binding data is shown in *D*. The *thick black lines* below the Httex1 sequence denote the binding region identified by peptide binding, whereas the *green* (or *green* and *magenta*) bars reflect the EPR results. The *green bars* for PHP7–PHP9 indicate the main binding region obtained by EPR, but sliding along the P1 and P2 is likely. The *magenta spots* for PHP5 and PHP6 symbolize the residues on the hydrophobic surface of the α-helix. The peptide binding experiments indicate that as few as five consecutive prolines (only a small subset of prolines present in P1 or P2) can be sufficient for binding to PHP7–PHP9. The *solid black five-residue box* thus indicates this minimal binding region, whereas *adjacent shaded boxes* reflect potential alternate binding sites within P1 and P2. The binding epitopes for MW1 (polyQ), PHP1, PHP2 (in L17) and P90, and MW8 (in C-ter) are indicated at the *top* of the *panel*. *Points* and *bars* in *A* and *B* indicate the mean ± standard deviation of triplicate measurements. EPR, electron paramagnetic resonance; Httex1, huntingtin exon-1.

In the case of PHP7–PHP9, no binding could be detected for the ΔPRD deletion mutant, whereas binding was observed for the ΔN17 mutant (Fig. S3). The importance of the PRD for PHP7–PHP9 binding was also further supported by peptide binding experiments, where PHP7–PHP9 most strongly interacted with PRD-derived peptides that contained at least five consecutive prolines from either the P1 or the P2 region (Fig. 2*B*). Peptides corresponding to the L17 region did not exhibit detectable binding, despite being rich in proline and containing a stretch of three consecutive prolines. Intriguingly, even peptides with proline repeats that included C-terminal L17 sequences showed low binding, suggesting that the L17 region may not only fail to bind PHP7–PHP9, but it might even reduce the PHP7–PHP9 affinity of the adjacent proline repeats. This notion is consistent with the EPR data, which show more binding for the portions of the P1 and P2 regions that are farther from the L17 domain (Fig. 1*C*). Aside from the potential impact of the L17 residues, there was no clear requirement for essential flanking sequences that were required in addition to the polyproline repeats. For instance, one peptide recognized by PHP7–PHP9 contained nine glutamines followed by five prolines, whereas another strong binder lacked glutamines entirely (see *arrows* in Fig. 2*B*). Thus, five prolines alone appeared to be a minimal binding unit for PHP7–PHP9. Given that P1 and P2 contain 11 and 10 proline residues, respectively, it is likely that only a subset of the consecutive prolines in each region contributes to antibody binding at any one time. Consequently, it is likely that multiple bound states exist, where PHP7–PHP9 bind to different segments of P1 or P2, potentially even “sliding” along the proline repeats with a preference for residues farther away from the L17 region (see Fig. 3*D* for a schematic summary of the EPR and peptide binding data).

Since PHP5 and PHP6, unlike PHP7–PHP9, appeared to recognize a single, well-defined binding site on Httex1, we sought to obtain more detailed structural information for this interaction by means of nitroxide scanning experiments. Such experiments can provide information with respect to secondary structure by highlighting residues that are at buried or exposed sites, as well as by detecting poorly folded and highly dynamic regions (29, 30, 31). To this end, we utilized a previously generated library of spin-labeled N17 derivatives of Httex1(Q25), a less aggregation-prone variant that can readily be used in its cleaved form without the need for a thioredoxin fusion partner (32). As shown in Figure S4, the EPR spectra of spin-labeled sites in the N17 region underwent significant line broadening upon interaction with PHP5 and PHP6. This line broadening was indicative of immobilization, which can be conveniently quantified using the inverse of the central line width (30). When plotted as a function of residue number, the inverse central line width revealed a periodic oscillation from residues 4 to 18, where maxima (and minima) were spaced three to four residues apart (Fig. 3, *A* and *B*). This periodicity, which was observed for both antibodies, is indicative of an α-helix, which has 3.6 residues/turn (31). The labeling sites with the smallest inverse central line widths (strongest immobilization) fall onto the hydrophobic side of the N17 helix (*magenta*, Fig. 3, *A* and *B*), whereas those with the largest inverse central line widths (*green*, Fig. 3, *A* and *B*; least immobilization) fall onto the opposing, hydrophilic surface (Fig. 3*C*). Together, these data indicate that PHP5 and PHP6 contact the hydrophobic surface of an amphipathic α-helix formed by the N17 region and Gln18 (Fig. 3*C*). The length of this binding region is in reasonable agreement with the peptide binding data (see summary in Fig. 3*D*).

**Figure 4:**
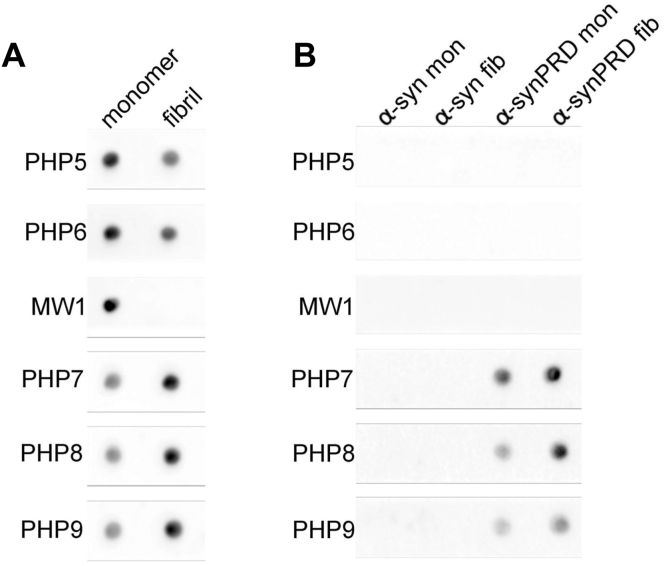
**Dot blot of antibody specificity toward monomers or fibrils of Httex1 and α-synuclein (α-Syn)–PRD chimera**. The PHP5–PHP9 and MW1 binding to Httex1(Q46) monomers and T-fibrils is shown in *A*, whereas the corresponding reactivity to α-Syn monomers and fibrils (*left*), and monomers and fibrils from α-synuclein–PRD chimera (α-SynPRD, *right*) are shown in *B*. Quantification of signal intensities using ImageJ (Fiji) shows PHP5 and PHP6 both have ∼1.4-fold preference for monomer *versus* fibrils, and PHP7–PHP9 have ∼2-fold preference for fibrils *versus* monomers. Httex1, huntingtin exon-1; PRD, proline-rich domain.

Having established that PHP5–PHP9 can bind Httex1 monomers, we next tested whether they also detect fibrils. To this end, we first used dot blots to compare PHP5–PHP9 antibody binding to Httex1(Q46) monomers and unbundled fibrils (also referred to as T-fibrils), which were previously found to be more toxic than bundled fibrils (18). Interestingly, PHP7–PHP9 exhibited stronger binding to fibrils over monomers, whereas PHP5 and PHP6 experienced a decrease in fibril binding (Fig. 4*A*). As a control, we used MW1, which binds to a linear epitope in the polyQ region (24, 26, 33, 34) that is accessible in the monomer but not the fibrillar state (Fig. 4*A*). To test whether the increased fibril binding of PHP7–PHP9 requires the presence of the Httex1 fibril core, we repeated the experiments with an α-synuclein–PRD chimera, in which the Httex1 PRD was C-terminally fused to the N-terminal fibril core containing regions of α-synuclein (35). None of the PHP or MW1 antibodies reacted with α-synuclein monomers or fibrils, demonstrating their specificity for huntingtin (Fig. 4*B*). PHP5 and PHP6 showed no reactivity to α-synuclein–Httex1 chimera because of the absence of the N17 epitope. In contrast, PHP7–PHP9 bound to the chimeric protein, and the binding was more pronounced with fibrils than with monomers. These data demonstrate that the enhanced fibril binding of PHP7–PHP9 does not require the Httex1 core region, and that the enhanced affinity for fibrillar states is likely a consequence of the increased local concentration of PRD bristles on fibril surfaces. It should be noted that dot blots are semiquantitative tools, only allowing relative comparisons of binding affinities between samples.

**Figure 5:**
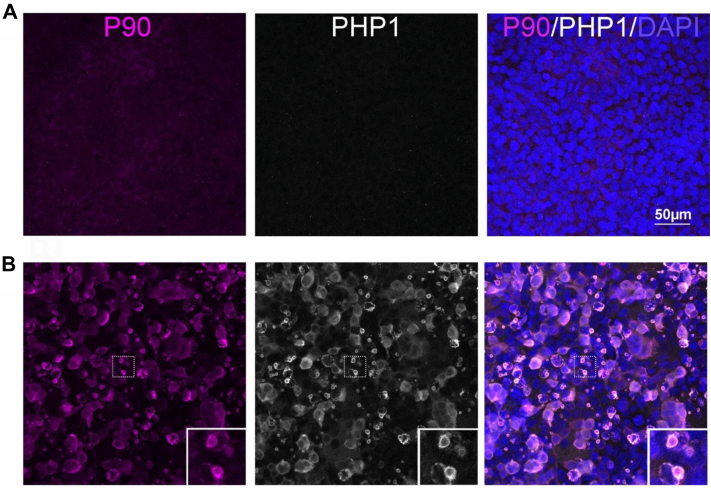
**P90 and PHP1 bind diffuse and aggregated Httex1(Q72) expressed in HEK293 cells**. *A*, control untransfected HEK293 cells incubated with P90 (*magenta*) and PHP1 (*gray*) antibodies. *B*, HEK293 cells transfected with Httex1(Q72) labeled by P90 and PHP1. *Insets* show antibody reactivity at the outer shell of the round inclusions. HEK293, human embryonic kidney 293 cell line; Httex1, huntingtin exon-1.

Following the *in vitro* characterization, we examined the reactivity of PHP5–PHP9 to Httex1 expressed in cultured cells or tissue from the R6/1 HD mouse model. To focus on potential conformational specificity, no epitope retrieval treatments were performed. Moreover, we sought to compare our results to those obtained from well-characterized control antibodies, such as PHP1. PHP1’s epitope is the peptide sequence QAQPLLPQP within the L17 region of the PRD (Fig. 3*D*, (23)). PHP1 has been shown to bind Httex1 monomers and with increased reactivity against fibrils of Httex1 *in vitro* and in cellular inclusions (23). However, distinguishing PHP5–PHP9 from PHP1 in immunofluorescence experiments is challenging because all these antibodies were generated in mice. To facilitate the comparison, we turned to the rabbit monoclonal antibody, P90, raised against the C-terminal neoepitope of Httex1 (AEEPLHRP-OH, Fig. 3*D*). Costaining of P90 and PHP1 in untransfected human embryonic kidney 293 (HEK293) cells shows little or no background (Fig. 5*A*). Both antibodies exhibited bright immunofluorescence in cells expressing Httex1(Q72) with a high degree of overlap (Fig. 5*B*). Both antibodies show cells that fall into two different staining patterns: one population shows a diffuse cytoplasmic signal, presumably from monomers and potentially smaller oligomers, and the other shows round inclusions, with antibody reactivity around their outer shell (Fig. 5*B*, *insets*). The reactivity at the shell of the Httex1(Q72) round aggregates using different Htt antibodies has been noted previously (36) and may reflect poor antibody access to the interior of the core, epitope masking, or the absence of an antibody epitope. Having established that P90 and PHP1 show similar reactivities against Httex1(Q72), we used P90 in costaining experiments to compare the properties of PHP5–PHP9 antibodies in HEK293 cells expressing Httex1(Q72).

**Figure 6:**
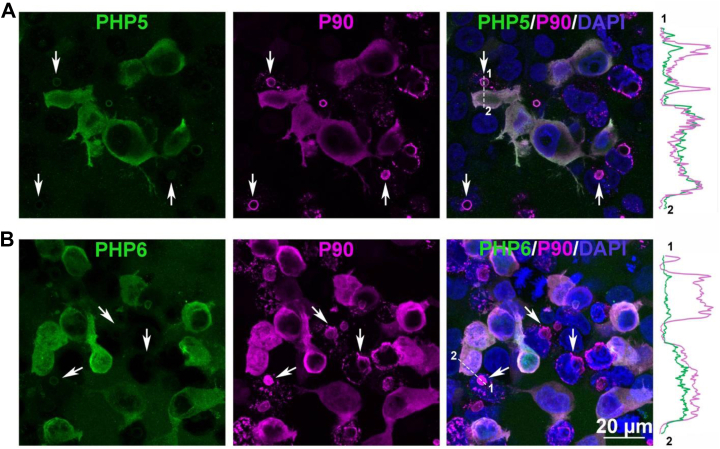
**PHP5 and PHP6 antibodies preferentially label diffuse Httex1(Q72)**. HEK293 cells transfected with Httex1(Q72) were labeled with (*A*) PHP5 and P90 or (*B*) PHP6 and P90. PHP5 and PHP6 signals are in *green* and P90 in *magenta*. The *right panels* show an overlay of both signals with DAPI (*blue*). *Arrows* point to round Httex1(Q72) inclusions. Plots of signal intensities for P90 (*magenta*) and PHP5 or PHP6 (*green*) along the *dashed lines* are measured in ImageJ (Fiji) and shown on the *right*. DAPI, 4',6-diamidino-2-phenylindole; HEK293, human embryonic kidney 293 cell line; Httex1, huntingtin exon-1.

**Figure 7:**
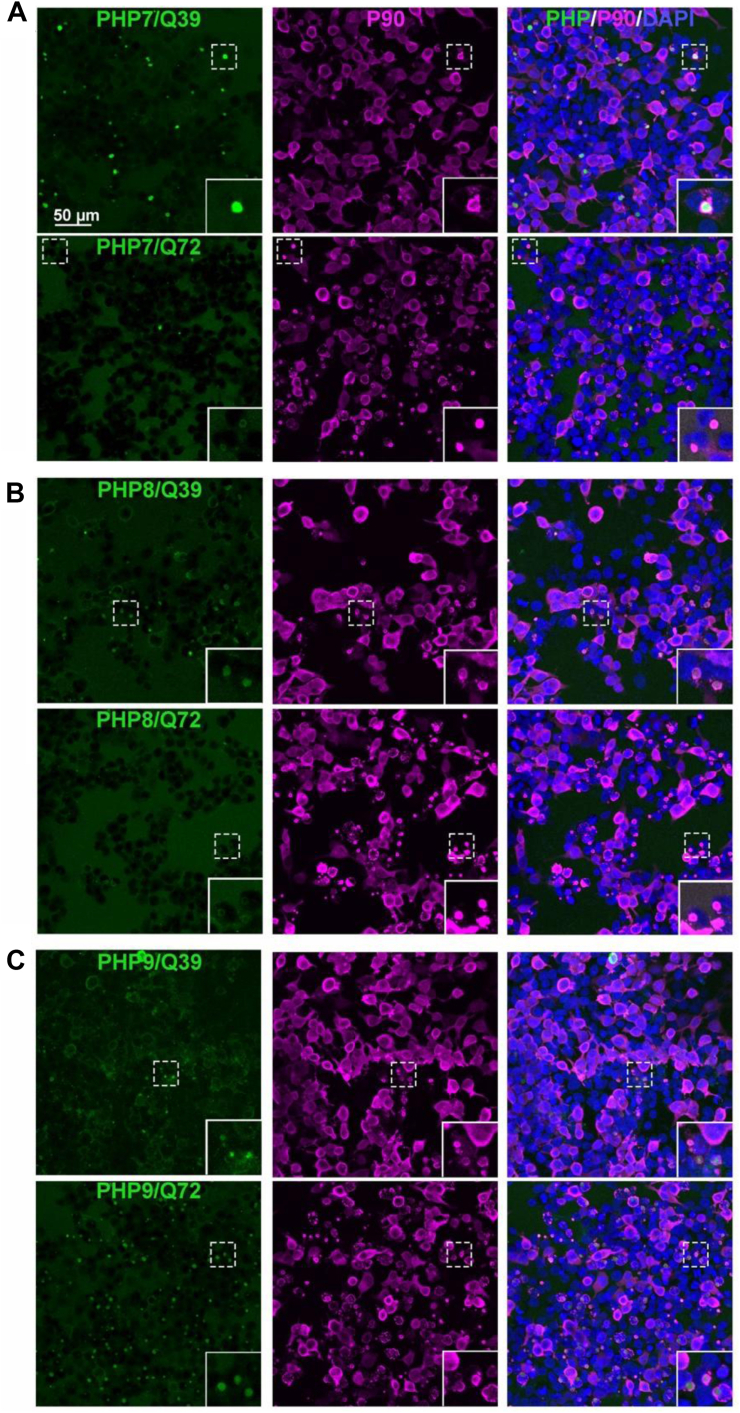
**PHP7, PHP8, and PHP9 antibodies preferentially bind Httex1 aggregates in HEK293 cells**. HEK293 cells transfected with Httex1(Q39) or Httex1(Q72) were stained with (*A*) PHP7, (*B*) PHP8, and (*C*) PHP9. PHP7–PHP9 signals are shown in *green*, and cells were costained with P90 (*magenta*). The *right panels* show both signals overlayed with DAPI (*blue*). *Insets* show magnified images of regions marked in each panel. DAPI, 4',6-diamidino-2-phenylindole; HEK293, human embryonic kidney 293 cell line; Httex1, huntingtin exon-1.

PHP5 and PHP6 immunofluorescence in HEK293 cells transfected with Httex1(Q72) and Httex1(Q39) (not shown) is similar, as both antibodies showed diffuse cytoplasmic labeling (Fig. 6). The round aggregates, highlighted by P90, stained poorly by PHP5 and PHP6 (Fig. 6, *arrows*). This is demonstrated in the plot of signal intensity profile for P90 (*magenta*) and PHP5, PHP6 (*green*) along the *dashed lines* (Fig. 6, *right panels*), where the aggregates show a higher intensity for P90. The staining pattern of PHP5 and PHP6 is highly specific, as low background staining was observed in nontransfected HEK293 cells (Fig. S5). These results are consistent with *in vitro* binding data showing similar binding specificity of PHP5 and PHP6 (Fig. 4) and suggest that the N17 epitope is more exposed in diffuse than aggregated Httex1(Q72). In contrast to PHP5 and PHP6, PHP7–PHP9, which show little background staining in nontransfected cells (Fig. S6), stain diffuse Httex1 poorly in HEK293 cells expressing Httex1(Q39) or Httex1(Q72). Instead, they have stronger reactivity against the aggregated state. Their reactivity is seen in round Httex1(Q39) and Httex1(Q72) aggregates (Fig. 7). These aggregates are identified by the P90-positive shell around these round inclusions (Fig. 7, *insets*). Intriguingly, the staining pattern of PHP7 and PHP8 for Httex1(Q72) is different from that of Httex1(Q39), as both antibodies only faintly recognize aggregates formed by Httex1(Q72) (Fig. 7, *A* and *B*, *bottom panels*). On the other hand, PHP9 shows more pronounced, uniform staining of both Httex1(Q39) and Httex1(Q72) round aggregates (Fig. 7*C*). These results indicate that PHP7–PHP9 have a strong preference for aggregated, rather than soluble, protein. Furthermore, despite having the same sequence epitope, staining properties of PHP7–PHP9 differ between Httex1(Q39) and Httex1(Q72) inclusions. In addition to differences in shell *versus* core staining, there also seems to be a difference in staining by P90 and PHP7–PHP9, as some aggregates only seem to be detected by P90 but not by PHP7–PHP9. Thus, significant structural heterogeneity of Httex1 aggregates exists within (rim *versus* interior) and between aggregates.

**Figure 8:**
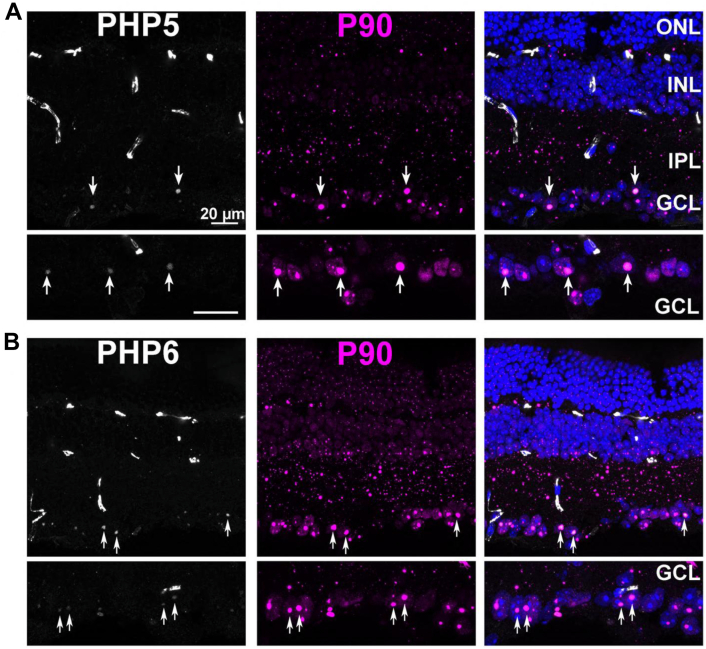
**PHP5 and PHP6 antibodies weakly label nuclear inclusions in R6/1 retinae**. *A*, retinal section from R6/1 mouse stained with PHP5. *B*, retinal section from R6/1 mouse stained with PHP6. Nonspecific labeling of retinal vessels is seen using the mouse PHP5, PHP6 antibodies. *Bottom panels* show magnified images of the ganglion cell layer (GCL). Sections were costained with P90 (*magenta*). *Arrows* show colocalization of PHP and P90 signals. INL, inner nuclear layer; IPL, inner plexiform layer; ONL, outer nuclear layer.

**Figure 9:**
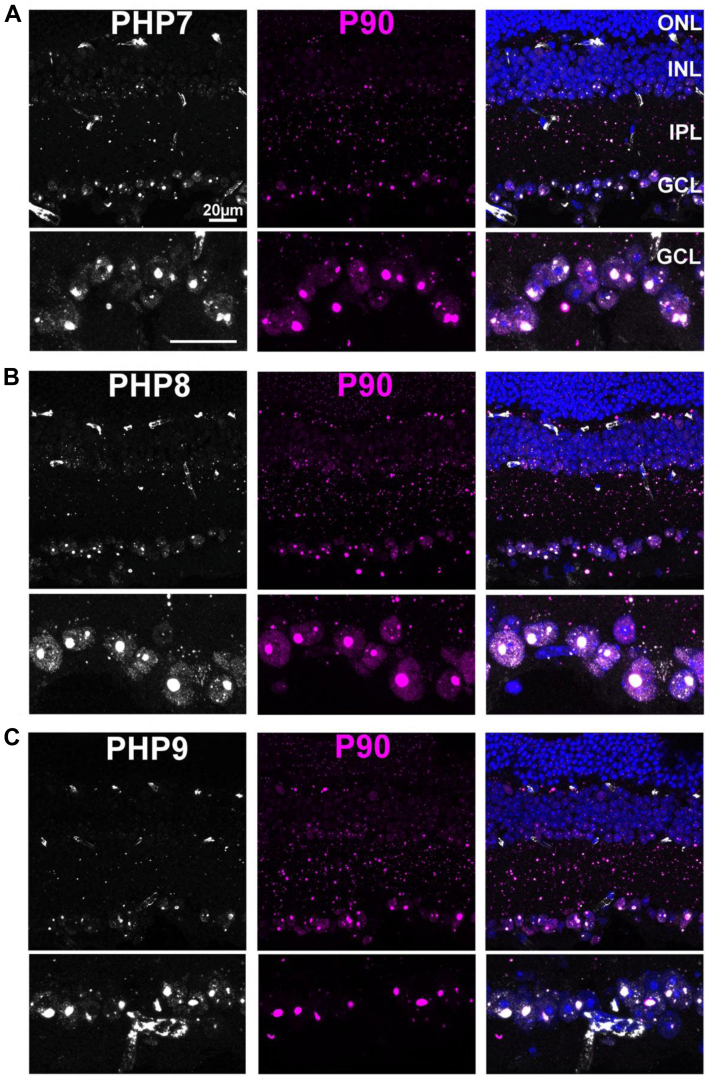
**PHP7, PHP8, and PHP9 antibodies strongly label Httex1 aggregates in all retinal layers**. Retinal sections stained with (*A*) PHP7, (*B*) PHP8, and (*C*) PHP9 antibodies. All sections were costained with P90. The scale bar represents 20 μm. Httex1, huntingtin exon-1.

Antibodies against different domains of huntingtin have been a resource for the detection of various forms of the protein in tissues from animal models and postmortem human brain. Toward this goal, we examined the ability of PHP5–PHP9 in the detection of pathogenic Httex1 in the R6/1 transgenic mouse model (4), using P90 as a point of comparison. First, the reactivity of P90 and PHP1 was compared in retinal sections from R6/1 mice (Fig. S7). We have previously shown that retinal pathology closely tracks that of brain pathology in these mice (37). Unlike the brain, an aged retina does not contain fluorescent lipofuscin granules, offering an advantage for immunofluorescence studies. Tissues from nontransgenic littermate control animals show little background for P90 and strong staining of retinal vessels by PHP1 because of the presence of endogenous mouse immunoglobulins but little elsewhere (Fig. S7*A*, *asterisks*). On R6/1 retinal sections, both P90 and PHP1 labeled Httex1 in all retinal layers, showing a high degree of overlap (Fig. S7*B*), similar to the results from HEK293 cells. Before staining with PHP5–PHP9, we confirmed that nontransgenic retinal tissue contained low background with these antibodies, except for the previously described nonspecific labeling of retinal vessels (Fig. S8, (37)). On the R6/1 retinal sections, PHP5 and PHP6 showed low fluorescence from intranuclear aggregates within ganglion cells (Fig. 8, *A* and *B*, respectively, *arrows* highlight representative intranuclear inclusions identified through P90 staining). Little or no diffuse cytoplasmic staining was observed, in contrast to HEK293 cells (Fig. 6). The low reactivity against Httex1 aggregates is consistent with the notion that the epitopes for PHP5 and PHP6 are not exposed, similar to the results obtained from HEK293 cells. Moreover, the weak diffuse staining suggests a low level of soluble monomeric Httex1 in R6/1 tissue. The preference of PHP7–PHP9 for Httex1 aggregates can be seen in R6/1 retinal sections (Fig. 9). Like P90, staining is observed in all retinal layers. At the magnified images of the retinal ganglion cell layer (*bottom panels*), large intranuclear inclusions are strongly labeled by PHP7–PHP9 (Fig. 9, *A*–*C*), and this reactivity overlapped with that of P90. Notably, the fluorescent signal is stronger in retinal sections than in HEK293 cells as the signals for PHP7–PHP9 appear equivalent to P90, unlike in HEK293 cells (Fig. 7). Together, immunofluorescence results from PHP5 to PHP9 on Httex1 aggregates further support the conclusion that the P1 and P2 epitopes are exposed in many types of aggregates, whereas the N17 epitopes are not.

## Discussion

Here, we present a comprehensive biophysical, biochemical, and immunocytochemical analysis of how PHP5–PHP9 interact with Httex1, and how these interactions facilitate the detection of different Httex1 conformers. Raised against recombinant proteins or peptides, the antibodies effectively recognize Httex1 in both cellular and animal models of HD, where they allow for the detection of soluble Httex1 and make it possible to distinguish between different types of aggregates.

In studies using recombinant proteins, we found that all antibodies can bind monomers and fibrils, but clear preferences exist for the two different groups of antibodies. PHP5 and PHP6 preferentially bound to the N17 region, which was more accessible in monomers than in fibrils, causing these antibodies to preferentially recognize the monomeric form of Httex1. In contrast, PHP7–PHP9 targeted the P1 and P2 proline repeats within the PRD, resulting in a preference for fibrillar aggregates. These conformational preferences were further validated by immunocytochemistry. In HEK293 cells and in R6/1 mouse tissue sections, PHP5 and PHP6 resulted in diffuse staining patterns with limited reactivity toward inclusions. In contrast, PHP7–PHP9 showed strong binding to Httex1(Q39) aggregates in HEK293 cells and R6/1 mice with little diffuse staining.

Nitroxide scanning experiments revealed that PHP5 and PHP6 bind to the hydrophobic surface of an amphipathic α-helix formed by the N17 region and Gln18. This surface also mediates numerous other interactions, as it is involved in the binding of Httex1 to lipids (32, 38), and other proteins, including intrabodies (39). Moreover, it also promotes Httex1 self-association into dimers and tetramers (10), which has mechanistic implications for binding to PHP5 and PHP6. Since PHP5 and PHP6 shield the hydrophobic surface of the N17, such antibody binding is incompatible with N17-mediated dimer or tetramer formation. Thus, we expect that PHP5 and PHP6 not only favor monomer over fibril binding, but that monomers should also be preferentially recognized over dimers or tetramers. Overall, PHP5 and PHP6 should be well suited for the detection of free Httex1 monomers in the cell.

**Figure 10:**
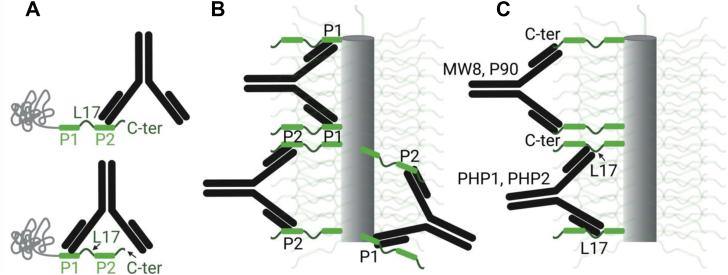
**Multivalent interaction of antibodies with the PRD in Httex1 fibrils**. *A*, a single PHP7–PHP9 antibody cannot bind to more than one epitope in a monomer at a time, because the distance between the binding regions on the antibody is much larger than the spacing between the epitopes in the PRD. *B*, PHP7–PHP9 and (*C*) PHP1, PHP2, MW8, and P90 can readily undergo bivalent interactions with Httex1 fibrils, which display a high density of PRD regions that radiate away from the fibril core. Note the different binding regions within the PRD for antibodies in *C*. Due to only one binding region for PHP1, PHP2, MW8, and P90, binding to monomers can also not be bivalent. In the case of PHP7–PHP9 in *A*, a second antibody might bind to give a 2:2 complex, which could be partially stabilizing. Created in BioRender. Lugo, J. (2025) https://BioRender.com/ebcpnvq. Httex1, huntingtin exon-1; PRD, proline-rich domain.

PHP7–PHP9 were raised against protofibrils of Httex1, yet neither of these antibodies recognized the polyQ core region. It is noteworthy that PHP7–PHP9, and other antibodies that have been used for the detection of Httex1 aggregates, target the PRD, even though it is the only region of Httex1 that essentially remains structurally unchanged during aggregation (17, 18). A likely explanation for this finding is that the polyQ and N17 regions are poorly accessible to antibodies in the fibril, limiting their utility for detecting conformational changes associated with aggregation. This notion is consistent with a recent computational study, which suggested that the PRD bristles make the core less accessible (40). In contrast, the PRD remains highly mobile and exposed in fibrils, making it readily accessible for antibody binding. So, why do PHP7–PHP9 prefer to bind fibrils over monomers? PHP7–PHP9 are bivalent antibodies, each with two antigen-binding sites typically spaced ∼130 Å apart (41). In monomeric Httex1, the P1 and P2 epitopes are separated by the L17 region, which spans at most ∼53 Å, even if one assumes a fully extended polyproline-II helical structure (17 residues × 3.1 Å per residue). This distance is too short to allow for simultaneous binding of both antibody arms to a single Httex1 monomer (Fig. 10). In contrast, Httex1 fibrils are decorated by a high density of outward-facing PRD "bristles," which are expected to facilitate bivalent interactions with PHP7–PHP9 (Fig. 10*B*). In addition, it is also possible that an antibody could promote fibril bundling (25) by simultaneously binding to bristles from two different fibrils. All these interactions would greatly enhance the binding affinity of PHP7–PHP9 for fibrils, and analogous arguments can be made for other PRD-binding antibodies (Fig. 10*C*). The notion that PRD bristle density promotes binding to PHP7–PHP9 is further supported by our α-synuclein–PRD chimera, which also yielded enhanced fibril over monomer binding. Thus, we propose that PRD binding antibodies (including PHP1, PHP2, P90, and MW8) exhibit preferential fibril binding because of their high density of readily available PRD bristles, rather than specific conformations imparted by the fibril core.

The present panel of antibodies points to a significant amount of heterogeneity within and between puncta. The heterogeneity within puncta is illustrated by PHP7–9 staining the interior of Httex1(Q39) aggregates, whereas P90 and PHP1 preferentially stain the halo region of the aggregates. Such heterogeneity is further supported by electron microscopy studies, which have shown that Httex1(Q39) aggregates are less dense and lack a dark shell structure exhibited by Httex1(Q72) inclusions (36). The heterogeneity between aggregates in HEK293 cells is highlighted by the fact that not all aggregates detected by P90 are also stained by PHP7–PHP9, even though all antibodies recognize epitopes in the PRD. On the other hand, while PHP7 and PHP8 reacted weakly with Httex1(Q72) aggregates in HEK293 cells, they both robustly labeled nuclear inclusions in R6/1 retina. These findings highlight that the panel of PRD-binding antibodies may be uniquely suited to distinguish between the different aggregate types because the respective epitopes are not equally accessible. The reason for how the different antibodies detect heterogeneity in the aggregates is not clear, but it would be interesting to find out what factors control the accessibility and to determine whether different types of aggregates might correlate with different degrees of toxicity.

In summary, our results indicate that targeting different regions of Httex1 can impart different degrees of selectivity toward monomeric or aggregated species and inform the design of different diagnostic and potential therapeutic binders. In some cases, it may be preferable to target smaller soluble species, which might be easier to degrade in the cells. In those cases, N17 or polyQ binders might be preferable. Conversely, targeting of larger aggregates might benefit from PRD binders.

## Experimental procedures

### Expression and purification of recombinant proteins

#### Httex1

Httex1 and cys mutants used for spin labeling were expressed in *Escherichia coli* as a thioredoxin fusion protein (Trx-Httex1) as described previously (7, 13, 18). As indicated in the text, some experiments used the fusion protein, whereas the thioredoxin tag was cleaved off in other experiments. The sample preparations were identical, except for the additional enterokinase cleavage step used for generating untagged Httex1 (13, 18). Protein concentrations of the fusion protein were determined *via* absorption at 280 nm because of the presence of Trp residues in thioredoxin. In addition, concentrations of spin-labeled derivatives were obtained by EPR using double integration of the EPR spectra relative to those of a standard of known concentration. The N- and C-terminal truncation mutants were purified using identical methods and used as thioredoxin fusion proteins without any spin labeling.

#### Alpha-synuclein–PRD chimera

The coding sequence of the PRD domain of Httex1 was cloned in frame with the synuclein complementary DNA, encoding the first 108 N-terminal amino acids into a pET28a(+) bacterial expression vector, in frame with a His-tag. This newly generated αSyn–PRD construct was expressed in *E*. *coli* BL21 (DE3) cells by inducing the culture at 0.5 to 0.6 absorbance with 1 mM isopropyl-1-β-d-galactopyranoside at 18 °C for 24 h. The bacterial pellets were harvested by centrifugation at 4000*g* for 15 min at 4 °C.

For protein purification, bacterial pellets were resuspended in ice-chilled 50 mM Tris–HCl, pH 7.4, containing 150 mM NaCl (Tris-buffered saline [TBS]). Bacterial cells were lysed using pulse sonication on an ice bath. The lysate was cleared by centrifugation at 30,000*g* for 20 min at 4 °C. The supernatant was transferred into a prechilled beaker, kept on an ice bath, and stirred gently with a magnetic stirrer bar. An ice-chilled saturated ammonium sulfate solution was slowly added to the protein solution to achieve a final 48% ammonium sulfate saturation. Under these conditions, the protein precipitates within 10 min. The top 50% of the solution was gently decanted, whereas the bottom 50% solution containing protein precipitate was centrifuged at 3000*g* for 30 min at 4 °C. The supernatant was discarded, and the pellet was resolubilized in TBS. The protein solution was then fractionated through a C4 reverse-phased HPLC column under the gradient of acetonitrile containing 0.1% TFA. The purified fraction was stored in a lyophilized condition until further use.

### Antibodies

PHP5–PHP9 were obtained as ascites solutions as described in our prior publication (22). Unless otherwise indicated, antibodies were purified using Protein A IgG purification kits (Thermo Scientific), according to the manufacturer’s guidelines. Concentrations were determined using a Bicinchoninic Acid Protein Assay Kit (EMD Millipore Corp). In addition, we also obtained purified PHP5–PHP9, PHP1, and P90 from CHDI.

### Spin labeling and EPR experiments

Trx-Httex1 was spin labeled using a 10-fold molar excess of MTSL spin label (Toronto Research Chemicals, Inc) to give the new side chain R1 as described (31). Free, unreacted spin label was washed away, and the spin-labeled protein was further purified according to previously described protocols (13, 18).

Nitroxide scanning experiments were designed to determine the local secondary structure of Httex1(Q25) bound to PHP5 or PHP6. To this end, spin-labeled derivatives of Httex1(Q25) were generated, where one spin label was introduced at consecutive sites between residues 2 and 21, one amino acid at a time. The derivative containing a label at position 15 (15R1) was not included because of difficulties in obtaining tag-free protein. For the EPR experiments, 10 μM of spin-labeled Httex1(Q25) derivatives were incubated with 10 to 20 μM of PHP5 or PHP6 in 50 mM Tris, pH 7.4, buffer containing 150 mM NaCl. A higher concentration of antibody (77 μM) was used for the 7R1 derivative, which had reduced PHP5 and PHP6 binding. To facilitate the line shape analysis and obtain a clean spectrum for the bound state, residual sharp lines from monomeric, unbound protein were subtracted using the spectrum of the protein in the absence of antibody.

For the coarse mapping of the PHP5–PHP9 binding domains in Figure 1, Trx-Httex1(Q46) was used. Labels were introduced in different regions of the protein, but, unlike in the nitroxide scanning experiments, they were typically spaced ∼5 to 10 residues apart. Spin-labeled Trx-Httex1(Q46) (10 μM) was added to 5 μM or 0 μM of PHP5–PHP9 in ascites buffered with 20 mM sodium phosphate, pH 7.4, 150 mM NaCl. Spectral differences caused by the presence of the antibody indicated binding interactions, which were quantified using the ratio of the EPR spectral amplitudes observed in the presence or the absence of the antibody. In the case of PHP5 binding to 11R1, some spectral components from the unbound, monomeric protein were subtracted. In all cases, spin-labeled samples were loaded into borosilicate capillaries (0.6 mm inner diameter × 0.84 mm outer diameter; VitroCom), which served as sample holders for the EPR experiments. EPR spectra were obtained using an X-band Bruker EMX spectrometer equipped with a Bruker ER4119HS resonator (Bruker Biospin). EPR spectra were recorded at an incident microwave power of ∼12.6 mW at room temperature. A scan width of 100 G and a modulation amplitude of 1.5 G were used for all EPR spectra obtained. The recorded spectra were spin-normalized through double integration.

### Dot blots

Samples as specified in the figure legends were blotted onto nitrocellulose membranes. To reduce nonspecific binding, 5% bovine serum albumin (BSA) in TBS with Tween-20 (TBS-T) was added and washed using 0.1% BSA in TBS-T. Primary antibodies were added in 0.1% BSA in TBS-T at a 1:3000 dilution and incubated at 4 °C overnight, followed by washing with TBS-T. Secondary anti-mouse IgG antibody was then added at a 1:3000 dilution for 30 min at room temperature and washed using TBS-T with a final single wash using TBS only. Binding was determined using ECL Western Blotting Detection Reagents (Cytiva-Amersham) with a G:Box Chemi-XX6 GENESys apparatus.

To determine the epitopes recognized by PHP5–PHP9, we utilized arrays of dot blots that contained overlapping 14-mer peptides synthesized from the first 91 amino acids of Httex1(Q23). That is, the first dot contains the peptide corresponding to amino acids 1 to 14, the second dot contains the peptide corresponding to 4 to 17, the third dot contains the peptide corresponding to 7 to 20, and so forth. The experiments were performed as first described for the MW series of antibodies (24).

### Fibril formation of Httex1

Httex1(Q46) T-fibrils were prepared using a protocol similar to what has been previously described (18). Httex1(46Q) fibrils were grown at a concentration of 20 μM in 20 mM Tris, 150 mM NaCl at 4 °C for 1 day. T-fibril samples were harvested by ultracentrifugation at 4 °C for 90 min and subsequently redissolved in 0.5% TFA in H_2_O. Concentrations determined using CD measurements compared with known standards. The T-fibrils are simply referred to as fibrils in the text.

### Immunofluorescence of HEK293 cells and R6/1 retinal sections

HEK293T cells were seeded onto a 24-well plate with poly-d-lysine (0.1 mg/ml)–coated coverslips. At 70% confluency, cells were transfected with 1 μg of Httex1(Q72 or Q39) expression plasmid using polyethylenimine at a ratio of 1:3 according to the manufacturer’s protocol (TOCRIS). Twenty-four hours after transfection, cells were fixed for 10 min with 3.7% formaldehyde at room temperature and then rinsed twice in PBS. Fixed cells were incubated in blocking buffer (1% bovine serum albumin, 5% normal donkey serum, 0.1% Triton X-100 in 1x PBS) for 1 h at room temperature. An incubation buffer was made by diluting the blocking buffer 1:10 in PBS (0.1X blocking buffer). Antibodies in the 0.1X blocking buffer (mouse PH5–PHP9 at 3–4 μg/ml and rabbit P90 monoclonal antibody 11G2, Coriell HDCB CH03541 at 0.5 μg/ml) were applied to the coverslips and incubated overnight at 4 °C. The next day, cells were washed three times with PBS and incubated for 2 h at room temperature with Alexa Fluor 488 donkey anti-mouse (1:400 dilution, A32766; ThermoFisher), Alexa Fluor 594 donkey anti-rabbit secondary antibodies (1:400 dilution, A32754; ThermoFisher), and 4',6-diamidino-2-phenylindole (DAPI, 1 μg/ml; Vector Laboratories) diluted in 0.1X blocking buffer. Coverslips with cells were mounted onto glass slides with VECTASHIELD antifade mounting medium without DAPI.

Retinal sections from 35-week-old R6/1 (JAX stock #006471) and nontransgenic littermate control mice were prepared as previously described (37). Briefly, the eyecups were sectioned along the superior–inferior axis using a cryostat (Leica CM3050S), and 12 μm sections were stored at −80 °C until use. Prior to antibody labeling, sections were washed in PBS (2 × 5 min) and incubated in blocking buffer (1% BSA, 5% normal donkey serum, and 0.3% Triton X-100 in PBS) at room temperature for 1 h, followed by overnight incubation at 4 °C with PHP5–PHP9 and P90 diluted in blocking buffer at concentrations listed above. The next day, sections were washed in PBS (3 × 5 min) and then incubated with Alexa Fluor–conjugated secondary antibodies (1:400 dilution; ThermoFisher Scientific) together with DAPI for 2 h at room temperature. Sections were washed in PBS (3 x 5 min) and mounted with VECNTASHIELD antifade mounting medium without DAPI. Immunofluorescence images of cells and tissue sections were acquired on a Zeiss LSM 800 confocal microscope.

## Data availability

Data are available within the article or its supporting information.

## Supporting information

This article contains supporting information.

## Conflict of interests

The authors declare that they have no conflicts of interest with the contents of this article.
