# Supplementary material for: Epitope-specific antibodies can distinguish between soluble huntingtin exon-1 and its diverse cellular aggregates[image]
Source: J Biol Chem. 2025 Dec 12;302(2):111048. doi: 10.1016/j.jbc.2025.111048 (PMC12816906; doi:10.1016/j.jbc.2025.111048)
Supplement: Supplementary Material [file mmc1.docx]

**Epitope-specific antibodies can distinguish between soluble huntingtin exon-1 and its diverse cellular aggregates**

Joshua Lugo, Hui Xu, Jeannie Chen, Ali Khoshnan, Ralf Langen


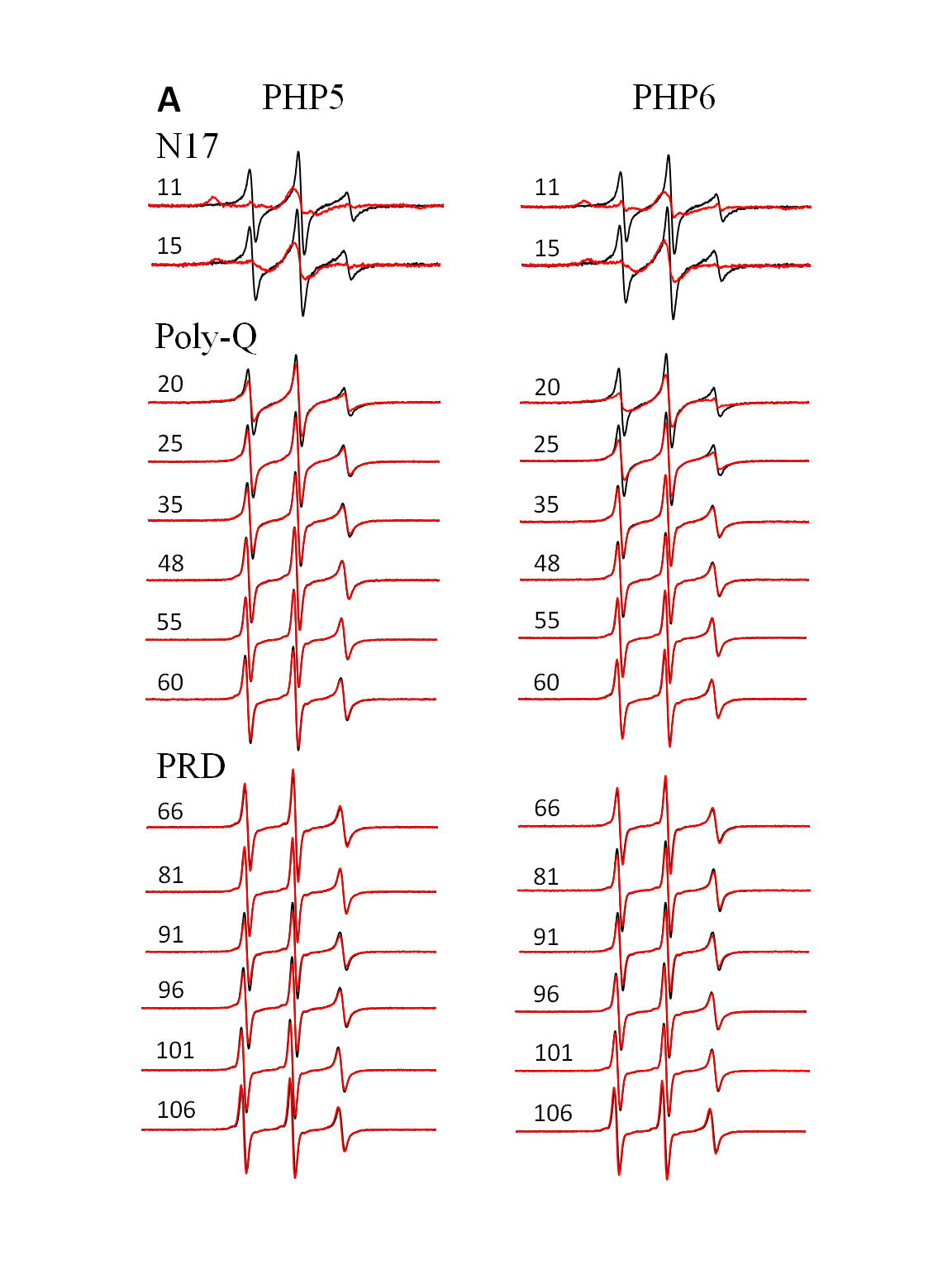


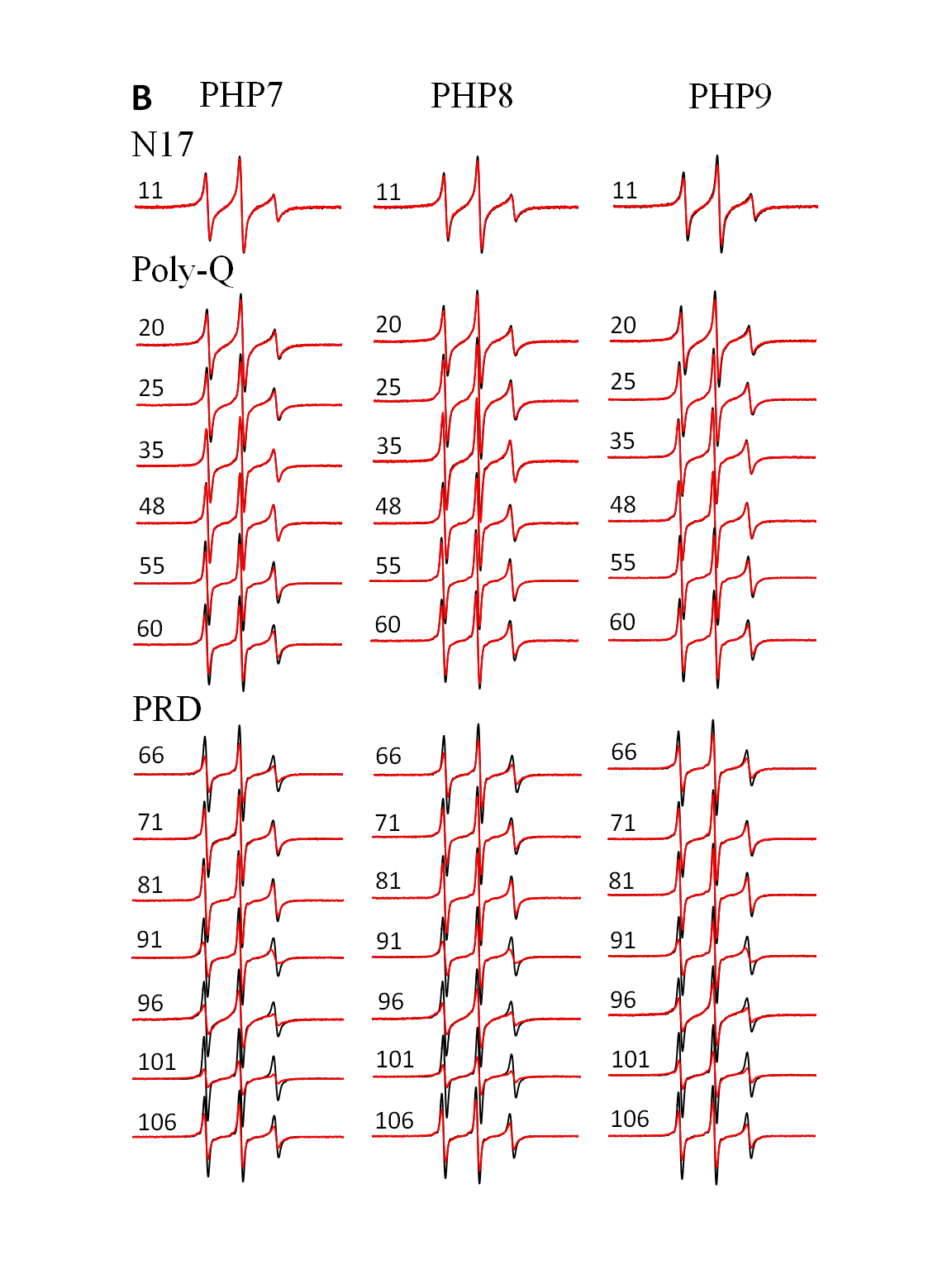


**Figure S1: PHP5-PHP9 binding regions revealed by EPR.** EPR spectra of spin-labeled Trx-Httex1(Q46) derivatives (10 μM) in the absence (black) or presence (red) of 5 μM antibody. Binding to PHP5 (left) and PHP6 (right) is shown in A, while binding to PHP7-PHP9 is shown in B. The scan width is 100 Gauss. The numbers to the left of the spectra indicate the residue, which was replaced with the spin-labeled residue R1. The spectra for the different derivatives were grouped by the domains where R1 was introduced. Spectra were obtained in triplicate and representative spectra are shown.


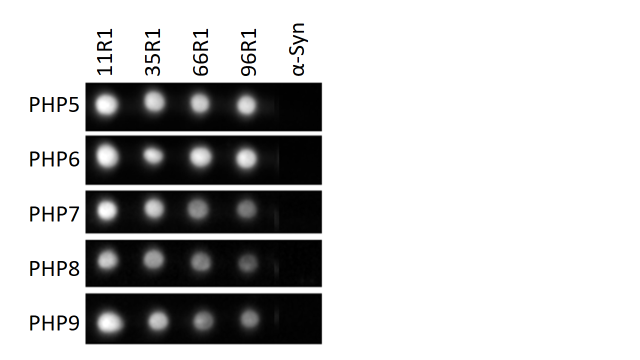


**Figure S2. PHP5-PHP9 binding to spin labeled proteins.** Dot blots showing PHP5-PHP9 binding to Trx-Httex1(Q46) derivatives that were R1 spin-labeled at positions 11, 35, 66, and 96. α-synuclein (α-Syn) was used as negative control.


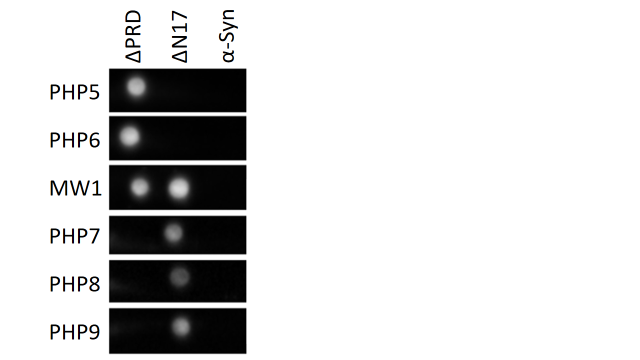


**Figure S3:** **Dot Blot of C-terminal (ΔPRD) and N-terminal (ΔN17) deletion mutants probed for PHP5-PHP9, and MW1 binding**. α-synuclein (α-Syn) is used as negative control and MW1, which binds to the polyQ served as positive control.


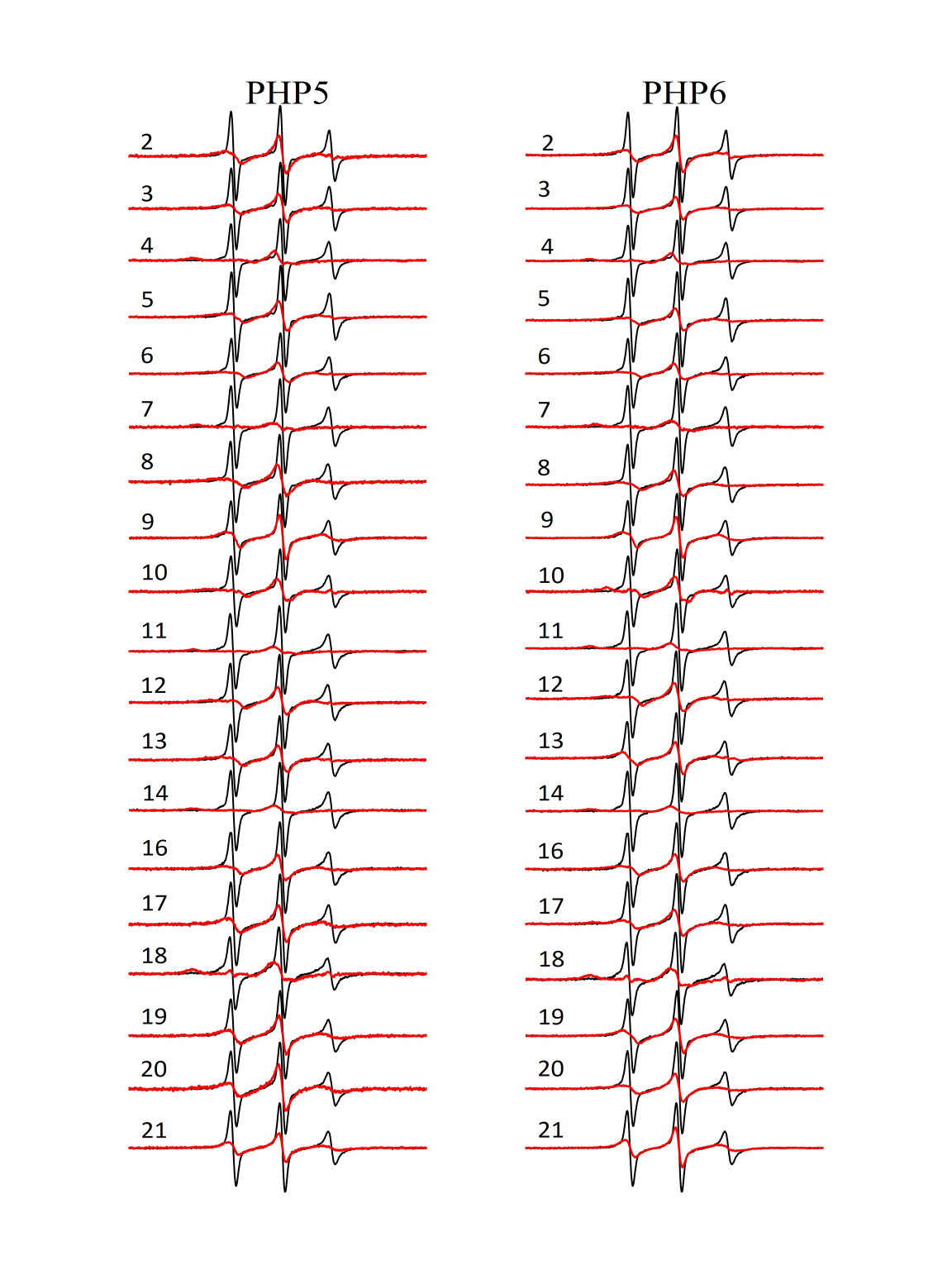


**Figure S4: X-band EPR spectra of spin-labeled Httex1(Q25) derivatives in the absence (black) or presence (red) of PHP5 and PHP6.** EPR spectra of 10 μM singly spin labeled Httex1(Q25) were obtained in the absence or presence of 10 to 20 μM PHP5 and PHP6. Due to incomplete binding, higher concentration (77 μM) was required for residue 7. The numbers to the left of the spectra denote the labeling sites where side chain R1 was introduced. The scan width is 100 Gauss. Spectra were obtained in triplicate and representative spectra are shown.

**
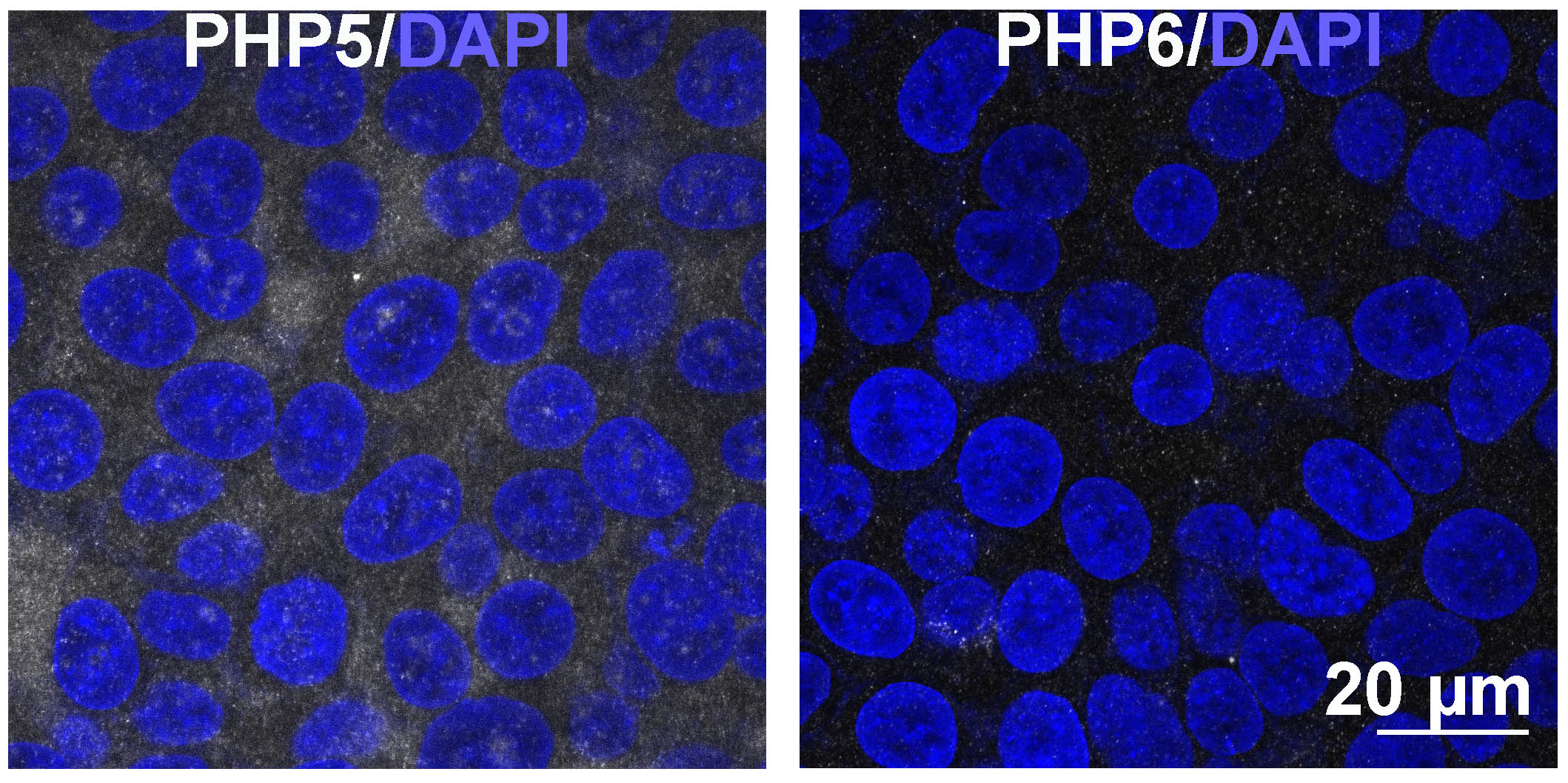
**

**Figure S5.** **PHP5 and PHP6 antibodies show low background staining in non-transfected HEK293 cells**.


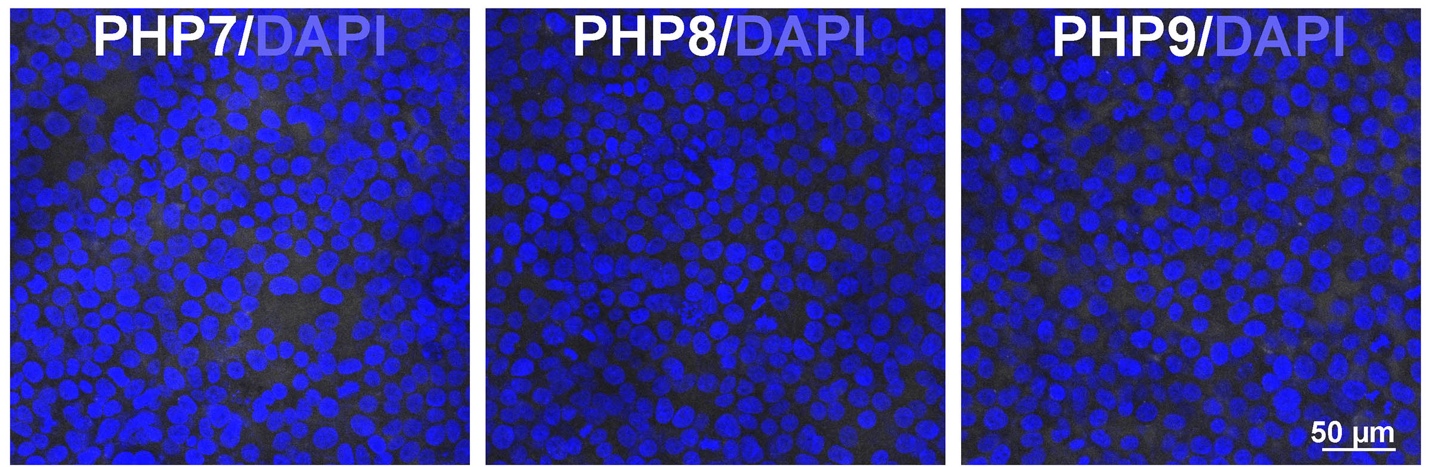


**Figure** S**6**. **PHP7, PHP8 and PHP9 antibodies show low background labeling in non-transfected HEK293 cells**.

**
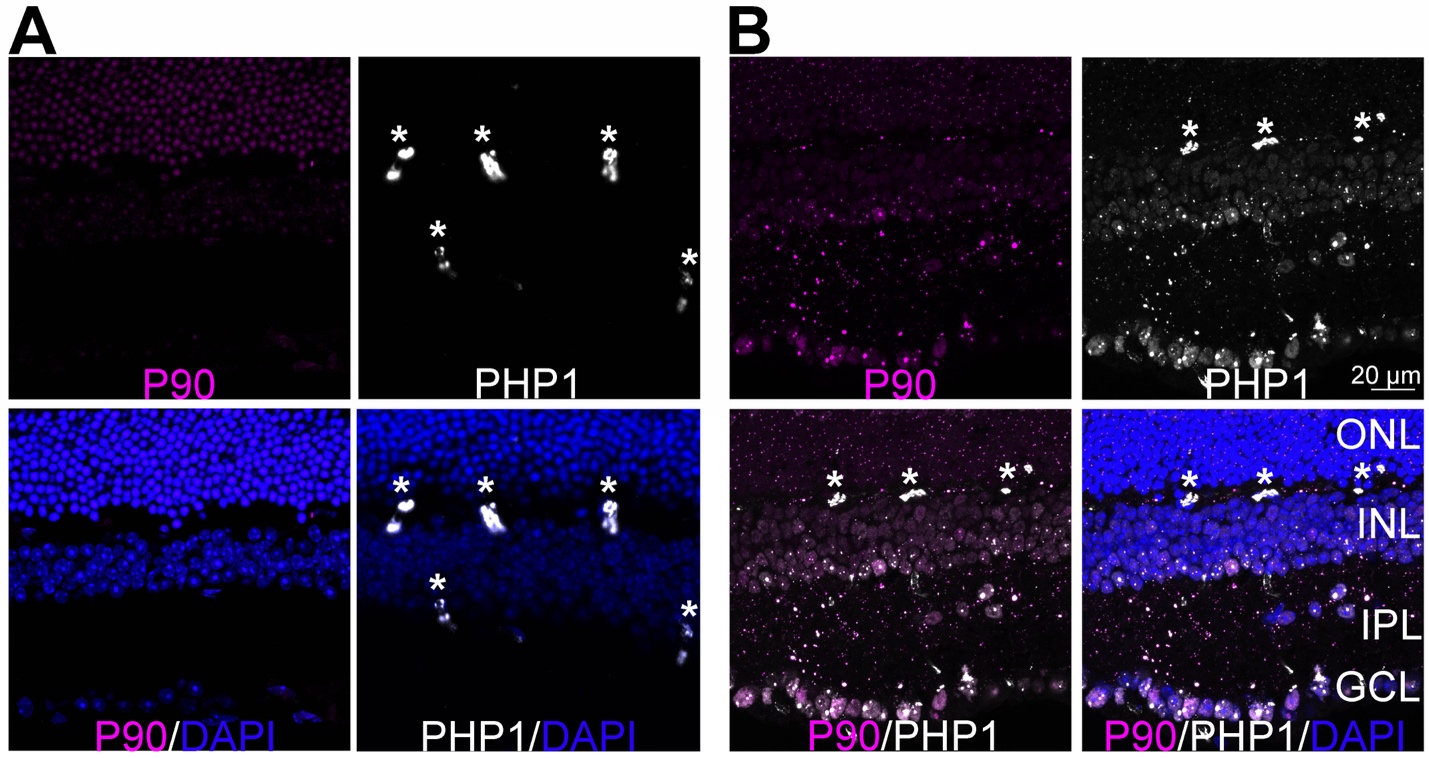
**

**Figure S7**. **Co-localization of P90 and PHP1 signals in R6/1 transgenic mouse retina**. A. Control non-transgenic retinal sections stained with P90 or PHP1. B. Retinal section from 34-week-old R6/1 mouse co-stained with P90 (magenta) and PHP1 (gray). Non-specific staining of retinal vessels (asterisks) can be seen in tissue incubated with PHP1, a mouse monoclonal antibody and fluorescently labeled secondary anti-mouse IgG antibody. ONL, outer nuclear layer; INL, inner nuclear layer; IPL, inner plexiform layer; GCL, ganglion cell layer.


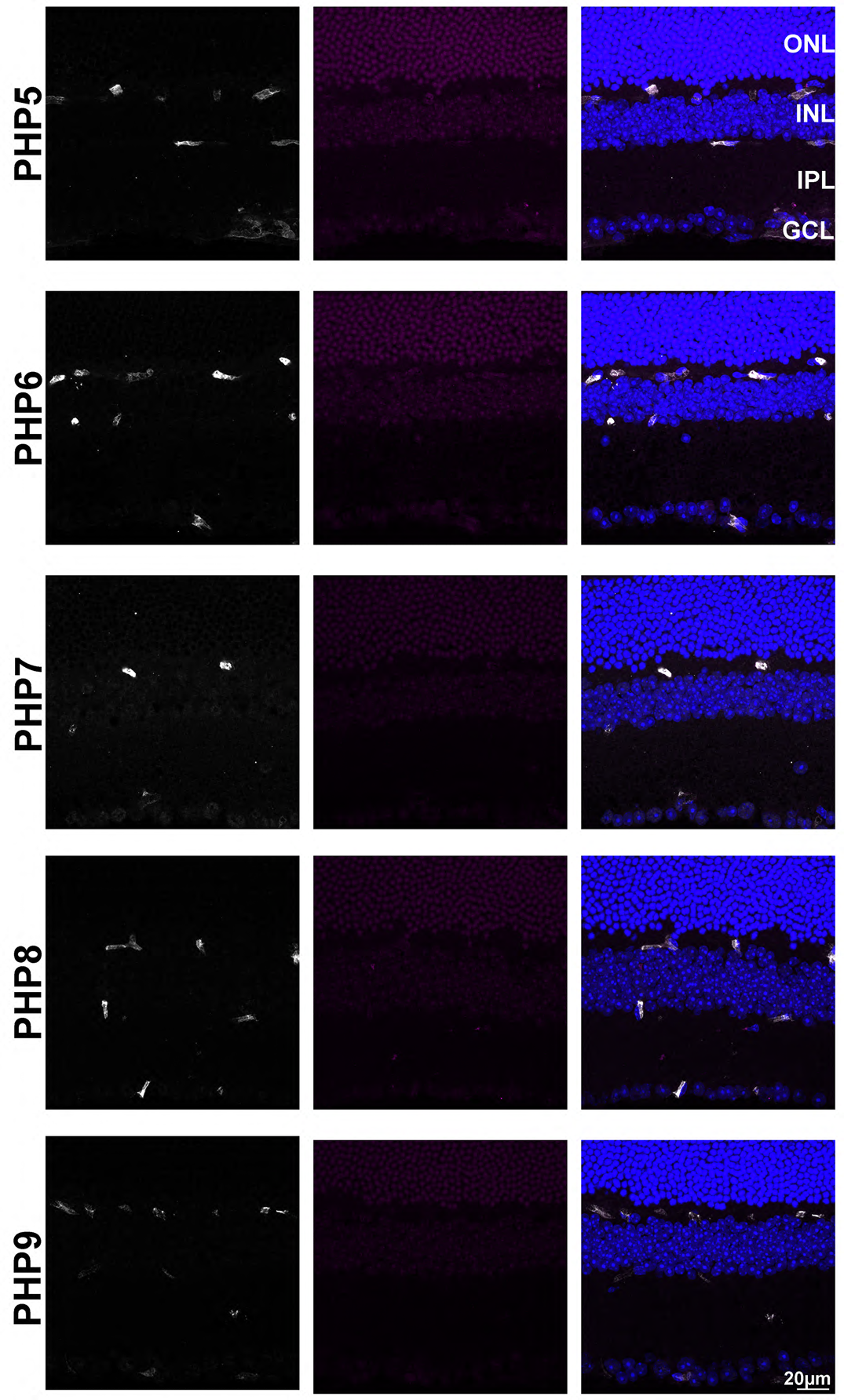


**Figure S8**. Retinal sections from non-transgenic R6/1 littermate control stained with PHP5-9 antibodies (gray), P90 (magenta) and DAPI (blue). ONL, outer nuclear layer; INL, inner nuclear layer; IPL, inner plexiform layer; GCL, ganglion cell layer.
